# INNOVATING CAREGIVER EDUCATION: INCLUDING COOPERATIVE EXTENSION IN RURAL CAREGIVING EDUCATION

**DOI:** 10.1093/geroni/igz038.2502

**Published:** 2019-11-08

**Authors:** Kristopher M Struckmeyer, Alex J Bishop, Gina Peek, Paula Tripp, Sarah Gordon

**Affiliations:** 1 Oklahoma State University, Stillwater, Oklahoma, United States; 2 Arkansas Tech University, Russellville, Arkansas, United States

## Abstract

Studies consistently report that caregivers under utilize resources, citing unawareness or inability to access programs as barriers to service utilization. Family and Consumer Science (FCS) educators within Extension have a unique blend of training that can help transform access to education for rural caregivers. Extension programming covers a wide range of topics, but few have implemented a curricular program or workshops to educate caregivers about caregiving issues. The current study involved a multi-state examination of innovativeness in caregiving program implementation among N = 216 FCS educators employed by the Cooperative Extension Service. Educators rated their perceptions of Extension’s receptiveness to change and personal factors. Results indicated that more urban areas (β = .19, p < .05), fewer years in their current position (β = -.23, p < .05), and greater leadership self-efficacy (β = .17, p < .05) predicted educator innovativeness to implement new caregiver education programming. However, when personal factors were added to the model, only years in current position (β = -.20, p < .05) remained significant. Subjective age (β = -.25, p < .01) and social support (β = .28, p < .01) were also found to significantly predict educator innovativeness. Despite previous research, these results indicate that personal factors may have a greater influence on educators’ innovativeness than organizational factors. These findings are critical when adopting and implementing a rural caregiver education programs through new organizational networks.

